# Antioxidant, Antimicrobial, and Insecticidal Properties of a Chemically Characterized Essential Oil from the Leaves of *Dittrichia viscosa* L.

**DOI:** 10.3390/molecules27072282

**Published:** 2022-03-31

**Authors:** Ibrahim Mssillou, Abdelkrim Agour, Aimad Allali, Hamza Saghrouchni, Mohammed Bourhia, Abdelfattah El Moussaoui, Ahmad Mohammad Salamatullah, Abdulhakeem Alzahrani, Mourad A. M. Aboul-Soud, John P. Giesy, Badiaa Lyoussi, Elhoussine Derwich

**Affiliations:** 1 Laboratory of Natural Substances, Pharmacology, Environment, Modeling, Health and Quality of Life (SNAMOPEQ), Faculty of Sciences Dhar El Mahraz, Sidi Mohamed Ben Abdellah University, Fez 30000, Morocco; abdelkrimagour1@gmail.com (A.A.); lyoussi@gmail.com (B.L.); elhoussinederwich@yahoo.fr (E.D.); 2 Laboratory of Plant, Animal and Agro-Industry Productions, Faculty of Sciences, University of Ibn Tofail (ITU), Kenitra 14000, Morocco; bioallali@gmail.com; 3 Department of Biotechnology, Institute of Natural and Applied Sciences, Çukurova University, Balcalı, 01330 Adana, Turkey; h.saghrouchni@gmail.com; 4 Laboratory of Chemistry, Biochemistry, Nutrition, and Environment, Faculty of Medicine and Pharmacy, University Hassan II, Casablanca 20000, Morocco; bourhiamohammed@gmail.com; 5 Laboratory of Biotechnology, Environment, Agri-Food and Health, Faculty of Sciences Dhar El Mahraz, Sidi Mohammed Ben Abdellah University, Fez 30000, Morocco; abdelfattah.elmoussaoui@usmba.ac.ma; 6 Department of Food Science and Nutrition, College of Food and Agricultural Sciences, King Saud University, P.O. Box 2460, Riyadh 11451, Saudi Arabia; asalamh@ksu.edu.sa (A.M.S.); aabdulhakeem@ksu.edu.sa (A.A.); 7 Department of Clinical Laboratory Sciences, College of Applied Medical Sciences, King Saud University, P.O. Box 10219, Riyadh 11433, Saudi Arabia; 8 Toxicology Centre, University of Saskatchewan, Saskatoon, SK S7N 5B3, Canada; jgiesy@aol.com; 9 Department of Veterinary Biomedical Sciences, University of Saskatchewan, Saskatoon, SK S7N 5B4, Canada; 10 Department of Integrative Biology, Michigan State University, East Lansing, MI 48824, USA; 11 Department of Environmental Sciences, Baylor University, Waco, TX 76798, USA; 12 Unity of GC/MS and GC, City of Innovation, Sidi Mohamed Ben Abdellah University, Fez 30000, Morocco

**Keywords:** antimicrobial, *C. maculatus*, *D. viscosa*, free radical, GC-MS, insecticidal characteristic, medicinal plants, volatile compounds

## Abstract

Since some synthetic insecticides cause damage to human health, compounds in plants can be viable alternatives to conventional synthetic pesticides. *Dittrichia viscosa* L. is a perennial Mediterranean plant known to possess biological activities, including insecticidal properties. The chemical composition of an essential oil (EOD) from *D. viscosa*, as well as its antioxidant, antimicrobial, and insecticidal effects on the cowpea weevil (*Callosobruchus maculatus*) were determined. Forty-one volatile compounds were identified in EOD, which accounted for 97.5% of its constituents. Bornyl acetate (41%) was a major compound, followed by borneol (9.3%), α-amorphene (6.6%), and caryophyllene oxide (5.7%). EOD exhibited significant antioxidant activity in all tests performed, with an IC_50_ of 1.30 ± 0.05 mg/mL in the DPPH test and an EC_50_ equal to 36.0 ± 2.5 mg/mL in the FRAP assay. In the phosphor-molybdenum test, EOD results ranged from 39.81 ± 0.7 to 192.1 ± 0.8 mg AAE/g E. EOD was active on *E. coli* (9.5 ± 0.5 mm), *S. aureus* (31.0 ± 1.5 mm), *C. albicans* (20.4 ± 0.5 mm), and *S. cerevisiae* (28.0 ± 1.0 mm), with MICs ranging from 0.1 mg/mL to 3.3 mg/mL. We found that 1 µL of EOD caused 97.5 ± 5.0% insect mortality after 96 h in the inhalation test and 60.0 ± 8.3% in the ingestion assay. The median lethal concentration (LC_50_) was 7.8 ± 0.3 μL EO/L, while the effective concentration in the ingestion test (LC_50_) was 15.0 ± 2.1 μL EO/L. We found that 20 µL of EOD caused a reduction of more than 91% of *C. maculatus* laid eggs.

## 1. Introduction

The increasing demand of the world population for food requires the cultivation of grains on a large scale. Unfortunately, almost one-third of world grain storage is damaged by pests [1]. *Callosobruchus maculatus* (F.) is considered one of the most important pests in stored grains, damaging chickpeas, green, black, and red grams, and cowpeas, mostly in Africa and throughout the tropics [2]. In addition to the economic damage caused by this insect, the qualitative and quantitative losses are related to larval penetration inside the grains [3]. In grains infested with this insect, a high content of moisture and ash was observed, while nutritional values, such as carbohydrate and crude fate levels, were decreased [4].

Resistance to synthetic pesticides, generated by their excessive use, has led researchers to search for natural alternatives that present less danger to the environment or the health of humans [5]. Recently, essential oils (EOs) have been considered as an alternative for pest control, and several aromatic and medicinal plants have shown excellent effects in the preservation of stocks of grains and in the control of pests [6,7].

Currently, problems of microbial resistance are becoming more and more serious, and microbial pathogens are developing mechanisms to resist the effects of antibiotics and antimicrobials and even of new drugs [8]. Based on a high-level scenario in a simulation model, antimicrobial resistance can lead to death rates that exceed 10 million by 2050 [9]. The excessive use of antibiotics and synthetic antimicrobial agents against infectious diseases has allowed several microbial strains to develop resistance against these substances. This has prompted researchers to search for new substances from various sources, especially medicinal plants, with the purpose to treat infectious diseases [10].

Excessive production of free radicals in the cells can lead to oxidative stress. Several diseases have been reported as the consequence of high levels of free radicals. The human body requires antioxidants to defend itself against the damage caused by free radicals. Plants are considered a natural source of antioxidants, e.g., polyphenols, terpenoids, flavonoids, which have been reported to possess high antioxidant activity. EOs are known for their antioxidant activity and their ability to reduce the formation of free radicals [5].

Morocco is known for its diversity of flora, especially aromatic and medicinal plants. For that reason, traditional medicine has been practiced in Morocco since antiquity [11,12], specifically, in the old medina of Fez, Al Quaraouiyine University, which is one of the oldest universities in the world and has always contributed to medical science [13]. A number of studies have been conducted on the various biological activities of plants originating in Morocco [14,15].

The family Asteraceae, which is comprised of 23,000 species in 1100 genera, is considered one of the most diverse families of flowering plants. Species belonging to this family are known to produce a variety of chemical compounds that are characterized by the presence of biologically active molecules, such as polyphenols and flavonoids. These species are widely studied for their antioxidant, antimicrobial, and insecticidal properties [16,17]. The genus *Inula* is comprised of more than 100 species, some of which, due to their therapeutic and medicinal effects, are used in traditional medicine in the Mediterranean region. After a taxonomic revision, the species *Dittrichia viscosa* L. known as false yellowhead (synonym, *Inula viscosa* L., local common name: Trehla or Magramane) was classified in the genus *Dittrichia* [18]. *D. viscosa* is a perennial herbaceous plant that grows in ruderal environments of the Mediterranean region and is considered a source of food for butterflies, caterpillars, and moths [19]. The results of several studies have demonstrated pharmacological and biological applications of *D. viscosa*, which was reported to have anticholinesterase, antibacterial, anti-5-lipoxygenase, and anti-tyrosinase activities [16]. It is used as a diuretic, expectorant, anthelmintic, and antianemic. In Moroccan traditional medicine, it is used as a cataplasm or poultice for rheumatic pain, for the treatment of bronchitis and tuberculosis, and also for its antitumor activity [15]. *D. viscosa* has also been used to treat injuries, wounds, bruises, and intestinal disorders [20]. Several reports on the chemical composition of the EO extracted from this plant have found various classes of terpenoids, such as sesquiterpenes and monoterpenes that are known to exhibit pharmacological and biological activities [21,22,23].

EOs are natural substances of plant origin, characterized by various biological properties, such as antimicrobial, antioxidant, and insecticidal activities, that can be used as alternatives to synthetic compounds against microbial resistance and for the control of pests [24]. The aim of this study was to identify the chemical composition of an EO extracted from *D. viscosa* leaves by the use of gas chromatography–mass spectrometry (GC–MS) and to evaluate the antioxidant activity and antimicrobial potential of this EO against some microbial pathogens, as well as its insecticidal effect on the cowpea weevil *C. maculatus* (F.).

## 2. Materials and Methods

### 2.1. Plants Material and Essential Oil Extraction

The *D. viscosa* (Asteraceae) specimens used in this study were collected between October and November, 2020, during the flowering season as well as in the post-flowering period in Fez, Morocco (34°03′04.2″ N, 5°01′25.4″ W). The collected material was identified by Prof. Amina Bari of the Laboratory of Biotechnology, Environment, Agrifood, and Health, Sidi Mohamed Ben Abdellah University. A voucher sample (DV20201214) was deposited at the herbarium of the Faculty of Sciences, Fez. The material used (leaf) was dried in the laboratory in ambient air and sheltered from light and moisture. An essential oil (EO) was extracted from 300 g of dry leaves by hydro-distillation for 4 h, in a Clevenger-type apparatus, with 1 L of distilled water in 2 L flask [25]. The EO was dried over anhydrous sodium sulfate, filtered, and stored at 4 °C.

### 2.2. GC–MS Analysis

The EO chemical composition was identified using gas chromatography (TRACE GC-ULTRA, S/N 20062969, Thermo-Fischer Scientific, Waltham, MA, USA) coupled with mass spectrometry (Quadrapole, PolarisQ, S/N 210729, Thermo Fischer Scientific, Waltham, MA, USA). The analysis was carried out using a capillary column (HP-5MS) with a length of 50 m, an internal diameter of 0.32 mm, and a film thickness of 1.25 µm. The temperature was set from 40 to 280 °C with an increase of 5 °C/min. The temperatures of the injector and of the detector (PolarisQ) were, respectively, 250 °C and 200 °C. Ionization was carried out in electron-impact mode (EI, 70 eV). The flow rate of helium, as a carrier gas, was set to 1 mL/min, with a split ratio of 1:40. We injected 1 µL of EO for analysis. The percentages of its constituents were determined, and the components were identified by comparison of their retention times with those curated in the library of NIST-MS Search Version 2.0.

### 2.3. DPPH Radical Scavenging Activity

In this test, the DPPH (2,2-diphenyl-1-picrylhydrazyl) radical scavenging activity method was used to evaluate the antioxidant activity of the EO [26]. An aliquot of 100 μL of EO and 750 µL of DPPH solution at 0.1 mmol were mixed. After 30 min in darkness, the absorbance was measured at 517 nm using a spectrophotometer (Perkin Elmer). The percent inhibition (PI%) of DPPH was calculated using Equation (1):

PI (%) = (Ac – As/Ac) × 100(1)

where PI (%) is the percentage of inhibition of DPPH, *Ac* is the absorbance of the control, consisting of methanol, and *As* is the absorbance of the EOD tested. The tests were performed in triplicate, and the inhibitory concentration of 50% (IC_50_) was determined (Equation (1). Butylated hydroxytoluene (BHT) and ascorbic acid were used as synthetic antioxidants for comparison.

### 2.4. Test of Total Antioxidant Capacity (TAC)

We mixed 1 mL of reagent solution containing 0.6 M of sulfuric acid, 28 mM sodium phosphate, and 4 mM ammonium molybdate with 100 µL of EOD. The incubation was performed in a water bath at 95 °C for 90 min. A spectrophotometer (Perkin Elmer, Shelton, CT USA) programmed at 695 nm was used to measure the absorbance, with a blank containing 100 µL of methanol instead of the EO [25]. The results were expressed using a calibration curve of ascorbic acid in milligrams of ascorbic acid equivalent per gram of extract (mg AAE/g E).

### 2.5. Ferric Reducing Antioxidant Power (FRAP) Assay

Briefly, 200 μL of EOD was added to 500 μL of phosphate buffer (0.2 M, pH 6.6) and 500 μL of potassium ferricyanide [K_3_Fe(CN)_6_] at 1%. The incubation was set at 50 °C for 20 min. Then, 500 μL of trichloroacetic acid (TCA) at 10% was added. The upper layer of the solution (0.5 mL) was added to 500 μL of distilled water and 100 μL of FeCl_3_ (0.1%). A spectrophotometer (Jasco v-530, Tokyo, Japan) was used to measure the absorbance at 700 nm. BHT and ascorbic acid were used for comparison. The results were expressed as EC_50_ (mg/mL) which is the effective concentration corresponding to 0.5 of absorbance [27].

### 2.6. Antimicrobial Activity

#### 2.6.1. Microbial Strains

The antimicrobial activity was determined against clinical microbes; four bacteria were chosen, three of which were Gram-negative, namely, *Escherichia coli*, *Klebsiella pneumoniae*, and *Pseudomonas aeruginosa*, and one was Gram-positive, i.e., *Staphylococcus aureus*. We also tested one fungus, i.e., *Candida albicans*, and one yeast, i.e., *Saccharomyces cerevisiae*. All microorganisms were provided by the microbiology laboratory at the university hospital center Hassan 2 in Fez, Morocco.

#### 2.6.2. Disk Diffusion Method

This assay was performed according to previously described methods [28]. This test measured the zone of inhibition around disks (6 mm in diameter) of Whatman paper number 1 that had been wetted with 10 µL of EOD and placed on the surface of Petri dishes, already filled with Mueller–Hinton (MH) agar or Sabouraud (SB) agar and inoculated with microorganisms (1 × 10^8^ to 2 × 10^8^ CFU/mL). After incubation for 24 h at 37 °C for bacteria and 48 h at 30 °C for the fungus and yeast, the antimicrobial activity was evaluated by measuring the inhibition zone around the disks in millimeters. Streptomycin (STR) (0.02 mg/disc), Tetracycline (0.02 mg/disc), Imazalil (0.02 mg/disc), and Fluconazole (0.02 mg/disc) were used to assess EOD potency against bacteria, fungi, and yeast relative to standard antimicrobials.

#### 2.6.3. Minimum Inhibitory Concentration (MIC) Assay

MICs were determined by use of the microdilution method [29]. First, 0.5 McFarland units of microbial inoculum was diluted at 1/1000. Antimicrobial standards (5 mg/mL) were prepared in sterile distilled water (1/10 *v*/*v*), and a dimethyl sulfoxide solution (DMSO) was used for the EOD. Inoculation was performed by depositing 50 µL of the culture medium in each well of the microplate, and 100 µL of each sample was deposited in the wells of the first column, which was used as a negative control for microbial growth. Microdilutions were carried out by transferring 50 µL from the wells of the first column to the second and so on, except for the last column, which was considered as a positive control for microbial growth. Finally, the inoculation was carried out by adding 50 µL of the microbial suspension in all the wells. The microplate was incubated for 24 h at 37 °C for bacteria and at 30 °C for the fungus and yeast. After incubation of the microplates, the growth of the microorganisms was revealed by a white spot below the wells, and for confirmation, 10 µL of 2,3,5-Triphenyltetrazolium chloride (TTC) (5 mg/mL) was added. Color change in the wells confirmed the presence of microbial activity.

### 2.7. Insecticidal Activity of EOD on C. maculatus

#### 2.7.1. Insect

The rearing of the species *C. maculatus* was carried out in the laboratory at 27 ± 1 °C, 65 ± 5% of relative humidity, and a photoperiod of 10:14 h light/dark.

#### 2.7.2. Assessment of EOD Toxicity by Ingestion of Chickpea Grains

The evaluation of the toxicity of EOD to *C. maculatus* was carried out according to the protocol described by Dutra et al., [30], where 20 g of chickpea seeds (*Cicer arietinum*) were infested with 10 *C. maculatus* (males and females; 0–48 h old) in glass containers (1 L) closed with plastic lids and lined with a transparent fabric. The EOD was added at four concentrations (1, 5, 10, or 20 μL/20 g) into the grains, which were stirred for 2 min. Containers without EOD were used as the negative control. The insect mortality (%) was calculated after 24, 48, 72, and 96 h, according to Equation (2):

(2) $$\mathrm{Pm}=100\times \frac{P0-\mathrm{Pn}}{100-\mathrm{Pn}}$$

where Pm = percentage of mortality, P0 = mortality observed in the trial, and Pn = mortality observed in the negative control.

Eggs were counted after 12 days from the start of the test, and the emerged insects were counted after 28 days. The percentage of reduction in the number of eggs and adults in the presence of EOD at different concentrations was calculated relative to the control according to Equation (3):

(3) $$\mathrm{Pl}=\frac{\mathrm{Ne}-\mathrm{Ni}}{\mathrm{Ne}}\times 100$$

where Pl = percentage of laying or reduction of emerged insects; Ne = number of eggs or insects hatched in the negative control, and Ni = number of eggs or insects hatched in the treatment.

#### 2.7.3. EOD Toxicity Using an Inhalation Test

The test was performed according to the protocol described by Allali and co-workers [31], where a small piece of cotton was placed into the bottom of a glass flask, and 10 *C. maculatus* individuals were placed in each flask. EOD was added to the cotton at different doses (1, 5, 10, 20 μL/L of air). All flasks were closed, and the dead insects were counted after 24 h, 48 h, 72 h, and 96 h. Insects were considered dead when no movement was noticed during 1 h. The comparison was made with a control sample (cotton without EOD). The percentage of mortality was calculated according to the formula used for the ingestion test.

### 2.8. Statistical Analysis

Data are expressed as means ± standard deviation (SD) of triplicate assays. Shapiro–Wilks and Levene’s test were used to test for the normality of the distributions and the homogeneity of variances, respectively. Analysis of the results was performed by GraphPad Prism software (version 9; Prism free trial) using one-way ANOVA followed by Tukey’s HSD test. A significant difference was considered at *p* < 0.05. The lethal concentrations LC_50_ and LC_95_ were determined by use of the probit method [32], using IBM SPSS Statistics v. 21. 

## 3. Results and Discussion

### 3.1. Yield and Chemical Composition of EO

Extraction of *D. viscosa* leaves by hydro-distillation produced an EOD with a specific odor and a yellowish to greenish color, with a yield of 0.09% (*w*/*w*). When the leaves, flowers, and aerial parts of *D. viscosa* where extracted, the yields of the EOs were 0.1% for the leaves and aerial parts, and 0.15% for the flowers [33]. In a separate study, the yields of EOs ranged from 0.03% to 0.07% [34]. In another study, the yield resulting from extraction of *D. viscosa* was 0.30% (*v*/*w*) [35]. This variation and differences in the yields of EOs among studies can be explained by edaphic effects on metabolites of plants but can also depend on the methods and solvents used for the extractions, as well as on the period of collection of the plants. All these factors can affect yields of EO [36,37].

**Figure 1:**
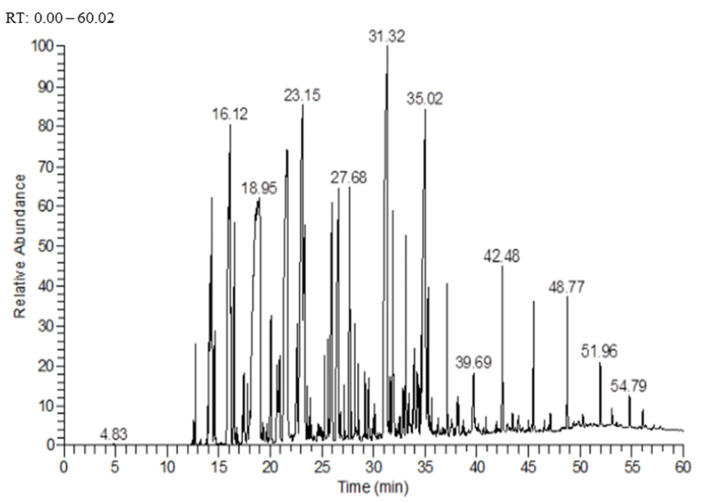
Chromatogram of the essential oil extracted from leaves of *D. viscosa*, presenting peaks with their retention times. Each peak represents the detector response for a different compound.

The GC–MS analysis revealed the presence of 41 volatile compounds in EO, corresponding to a total of 97% of the mass (Figure 1 and Table 1). The most abundant compounds in EO were bornyl acetate (41%), borneol (9.3%), α-amorphene (6.6%), and caryophyllene oxide (5.7%). The EO obtained in this study was mainly composed of monoterpenes and sesquiterpenes.

Bornyl acetate has been reported to possess several biological and pharmacological activities, such as antioxidant, anti-tumor, and anti-inflammatory activities [38,39,40]. EOs of other plants that contain bornyl acetate, were reported to have antimicrobial, antioxidant, and insecticidal properties [41,42,43]. Borneol, which was the second abundant compound in EO in this study, was also reported to possess antioxidant, antimicrobial, and insecticidal activities [44,45,46,47].

Several studies have reported the chemical composition of *D. viscosa* EOs, such as a study conducted on the chemical composition of the EO from Algerian *D. viscosa* [22], whose main compounds were 12-carboxyeudesma-3,11 (13) diene (29%), linolenic acid (7.8%), and pentacosane (5.4%). Another study conducted in Algeria [23] found that the main EO compounds were isocostic acid (59%) and fokienol (14.6%). Another study conducted on specimens from Spain revealed the presence of borneol (25 and 21%) and bornyl acetate (20 and 49.7%) as major compounds in two samples of *D. viscosa* EOs [20]. These results are similar to those reported here. Environmental factors, including genotype, method of extraction, region of harvest of the plant, organ used in the extraction, and time of collection, are the factors responsible for variations in the chemical composition of EOs [37,48,49]. Hence, the chemical composition of *D. viscosa* from different regions is an important factor determining its bioactivity. In EOs, even minor compounds can exert effects due to synergistic effects between chemical classes, and the presence of monoterpenes and sesquiterpenes in EOs promotes the bioactivities of other compounds.

### 3.2. Antioxidant Activity of EOD

EOD exhibited significant antioxidant activities. EOD showed good potential to reduce DPPH (IC_50_ = 1.30 ± 0.05 mg/mL) but was less potent compared to the antioxidants BHT (IC_50_ = 7.0 ± 0.1 µg/mL) and ascorbic acid (IC_50_ = 1.0 ± 0.1 µg/mL), as presented in Table 2. The ability of an antioxidant to scavenge free radicals is correlated to its capacity to exchange an electron or a hydrogen atom [50]. Results of other studies confirmed the potential of EOD [33,51], but the IC_50_ obtained in these studies was greater than the one we measured. A study carried out on the EOD of this plant using the DPPH test showed an IC_50_= 14.0 ± 0.4 µg/mL [52], indicating that this EOD oil was more effective than the one we produced and analyzed. Differences in results among studies can be explained by variations in the chemical composition of EOS. Bornyl acetate and its derivatives, including borneol, which are the most abundant chemical compounds of the EO studied here, were reported to possess good antioxidant activity [39,46].

Alternatively, when the FRAP assay was used to evaluate the ability of the EO to transform ferric iron Fe^3+^ to ferrous iron Fe^2+^ [53], moderate antioxidant activity (EC_50_ = 3.7 × 10^2^ ± 2.5 mg/mL), compared to BHT (EC_50_ = 1.3 ± 0.2 mg/mL) and ascorbic acid (EC_50_ = 0.76 ± 0.1 mg/mL), was observed (Table 2). The results of another study that was performed on the antioxidant activity of a *D. viscosa* EO using the FRAP assay found a reducing capacity of 24 mg Fe/mg oil [54].

**Figure 2:**
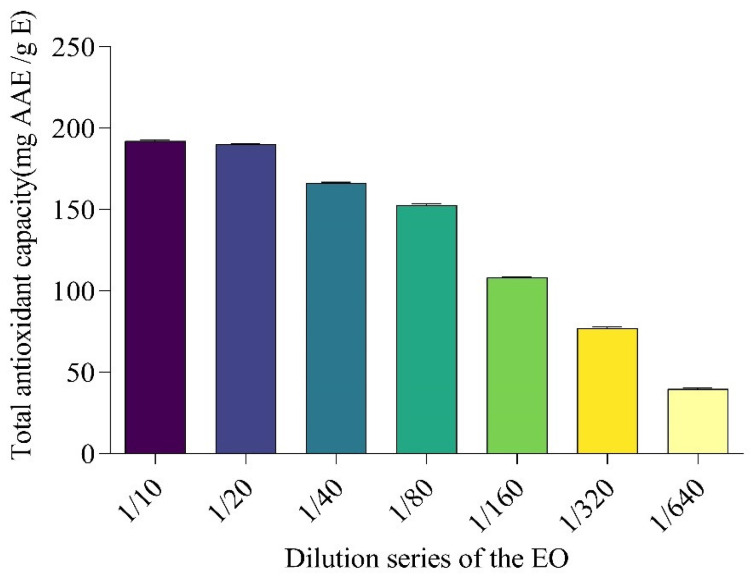
Total antioxidant capacity of the essential oil of *D. viscosa,* using a series of dilution (mg AAE/g E). Results are expressed as mean ± SD.

When the total antioxidant capacity of EO was evaluated using the phospho-molybdenum method [55], which is based on the transformation of Mo (VI) to Mo (V) in the presence of an antioxidant, with the appearance of a green to blue color and is expressed as mg ascorbic acid equivalent/gram of extract (AAE/g E), EO exhibited a significant antioxidant potential (Figure 2 and Table 3), corresponding to 192.1 ± 0.8 mg AAE/g E at minimal dilutions (1/10) and to 39.8 ± 0.7 mg AAE/g E at the greatest dilution (1/640).

### 3.3. Antimicrobial Activity

In the disc diffusion and microdilution plate tests, EO extracted from *D. viscosa* leaves exhibited antimicrobial activity against three Gram-negative strains, *E. coli*, *P. aeruginosa,* and *K. pneumoniae*, one Gram-positive strain, *S. aureus*, one fungus, *C. albicans*, and one yeast, *S. Cerevisiae* (Table 4 and Table 5).

In the disc diffusion test, EO was effective against *E. coli* (9.5 ± 0.5 mm), *S. aureus* (30.7 ± 1.5 mm), *C. albicans* (20 ± 0.5 mm), and *S. cerevisiae* (28 ± 1.0 mm) (Table 4). The positive control used in this study (streptomycin) was active only on *S. aureus* (9.5 ± 0.2 mm) with de minimis activity compared to the EO; therefore, the other strains showed resistance to this antibiotic. Minimum inhibitory concentrations showed that the EO was active on all microbes, with MICs ranging from 0.101 mg/mL to 3.25 mg/mL (Table 5). In contrast, the positive control (streptomycin) showed no activity on *P. aeruginosa*, while the EO exhibited a MIC of 1.6 mg/mL for the same microbe. The results of a previous study [56] on the antimicrobial activity of *D. viscosa* EO showed similar results, with MICs ranging from 20 ± 1.1 to 2.0 × 10^2^ ± 2.5 μg/mL. Extracts of this plant also exhibited an activity on food microbes [57]. In another study, compounds isolated from *D. viscosa* exhibited significant antimicrobial properties [58]. The results reported here are consistent with those of previous studies conducted in other countries on the ability of this plant’s EOs to inhibit microbial growth. This EO had a significant content of terpenoids, which are the major compounds responsible for antimicrobial activity, together with phenolic acid, flavonoids, and tannins [59]. In summary, EOs affect the integrity of microorganisms’ membrane causing some dysfunction in crucial mechanisms, such as nutrient transfer, metabolic functions, and growth regulation [60]. The variability observed in the antimicrobial activity of EOs is due to the diversity of chemical compounds, which differentially affect the permeability of the cell wall.

### 3.4. Insecticidal Activity

**Figure 3:**
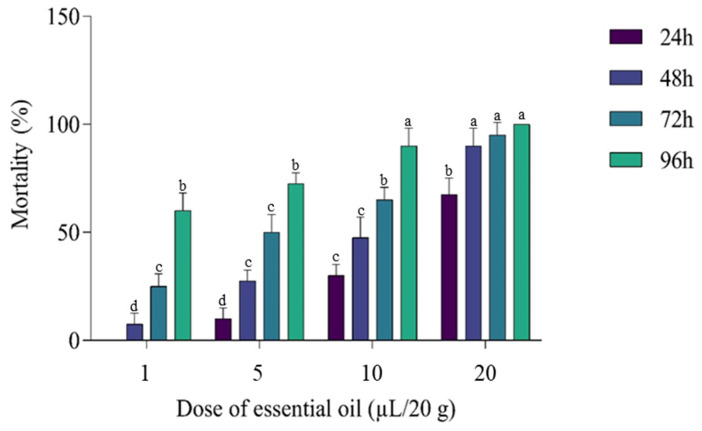
Mortality (%) of *C. maculatus* after ingestion of grains in presence of different doses of EO (µL EO/20 g). The test lasted four days, and the results were obtained at 24, 48, 72, and 96 h. All treatments had a significant effect compared with the control (0%). Bars with the same letters do not differ significantly (*p* < 0.05) (Tukey’s HSD test). Results are expressed as mean ± SD.

**Figure 4:**
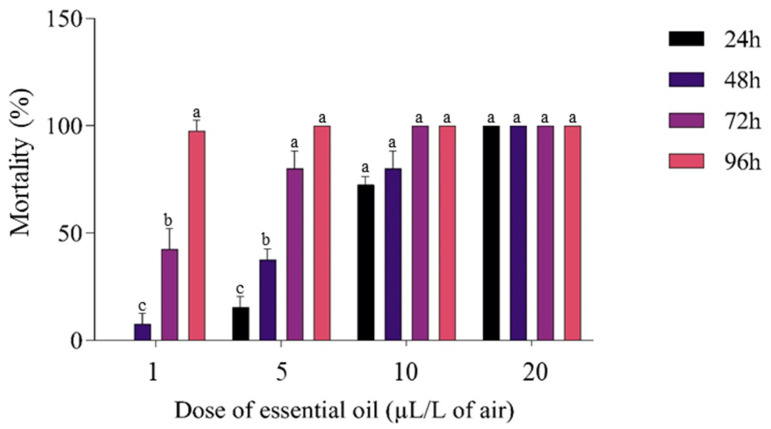
Mortality (%) of *C. maculatus* in the inhalation test caused by the exposure to the EO (µL/L of air). The test lasted four days, and the results were obtained at 24, 48, 72, and 96 h. All treatments had a significant effect compared with the control (0%). Column values with the same letters do not differ significantly (*p* < 0.05) (Tukey’s HSD test). The results are expressed as mean ± SD.

Ingestion of grains containing the EO of *D. viscosa* or vapors of the EO caused lethality in *C. maculatus* during the ingestion and inhalation tests (Figure 3 and Figure 4). The least concentration (1 μL EO/L) tested in the inhalation assay on *C. maculatus* caused 97.5 ± 5% mortality after 96 h of exposure, while this same dose caused only 60 ± 8.16% mortality in the test based on the ingestion of grains containing EO. The highest concentration (20 μL EO/L) caused 100% mortality via inhalation and 67.5 ± 7.5% via ingestion. The lethal concentration (LC_50_) in the inhalation test (7.8 ± 0.29 μL/L of air) was less than that observed in the ingestion test (14 ± 2.1 μL/20 g of grain) (Table 6). Note that the negative control (0 µL) did not cause any mortality (0%) during four days; a highly significant difference was observed between the negative control (0 µL) and other doses at *p* < 0.05.

Despite the early death of adults of *C. maculatus*, no concentration of EO completely prevented spawning in females. The number of eggs laid was inversely proportional to the concentrations of EO (Table 7). At the least dose (1 µL), the mean number of eggs laid per female was 94 ± 9.6, and a respective rate of reduction in laying of 49.28 ± 5.20% was observed compared to the control. At the highest concentration, the average number of eggs laid per female decreased sharply, reaching 15 ± 3.5, and the respective reduction in eggs laid was 92%. The number of eggs laid per *C. maculatus* female in the control containers was 1.8 × 10^2^ ± 2.3 × 10^1^. A significant reduction of 91% was observed for the rate of emergence at the greatest dose.

The EO of *D. viscosa* caused mortality of *C. maculatus* adults within 24 h. Exposure to EO via inhalation exhibited the strongest insecticidal effect, with the greatest concentration of 20 μL EO/L causing total (100%) mortality. The observed efficacy as well as the LC_50_, which was 7.8 ± 0.29 μL/L, could be due to the major compound in EO, i.e., bornyl acetate, or to combined effects of all compounds contained in EO. Furthermore, insecticidal activities of the most abundant compounds in EO, bornyl acetate and borneol, have been demonstrated in other species of beetles [45,61].

The chemical compounds contained in EOs affect the growth of insects through enzymatic processes and act as inhibitors of acetylcholinesterase and as mimics of octopamine. The inhibition of acetylcholinesterase in pests in stored products blocks the hydrolysis of acetylcholine. Monoterpenoids, such as bornyl acetate and borneol, are known for their acetylcholinesterase inhibition effects [62,63]. Therefore, the presence of such compounds in EO could cause the death of *C. maculatus* treated with the essential oil. The observed mortality might be due to one or a few compounds or to the combined effects of several chemicals. Effects can be caused by several other compounds, including g-aminobutyric acid or ligands of octopamine, tyramine, nicotinic, and acetylcholine receptors.

The results of other studies have shown that *C. maculatus* is a major beetle pest in stored chickpeas [64,65] and can cause significant damage to this stored product. Thus, efficacy of *D. viscosa* against *C. maculatus* supports the use of EOs as a natural alternative to synthetic pesticides to control *C. maculatus* in stored chickpeas. In summary, *D. viscosa* has insecticidal activity against several pests, including the African cotton leaf worm, the Egyptian cotton leaf worm, Mediterranean brocade (*Spodoptera littoralis*), a moth in the family Noctuidae, the green peach aphid, also called greenfly, the peach-potato aphid, (*Myzus persicae*), a small green aphid belonging to the order Hemiptera, and the bird cherry-oat aphid (*Rhopalosiphum padi*), an aphid in the superfamily Aphidoidea in the order Hemiptera [66]. The results of another study performed on the insecticidal activity of extracts of *D. viscosa* indicated insecticidal activity against the sawtoothed grain beetle (*Oryzaephilus surinamensis*), a beetle in the superfamily Cucujoidea, the confused flour beetle (*Tribolium confusum*), a type of darkling beetle, and the rice weevil (*Sitophilus oryzae*) [67]. Furthermore, compounds from *D. viscosa* have been reported to protect chickpea seeds against *C. maculatus* [68].

## 4. Conclusions

The EO evaluated in this study for its antioxidant and antimicrobial activities and its insecticidal efficacy against *C. maculatus* showed the presence of some important compounds such as borneol and bornyl acetate. Its chemical components provide the EO with the antioxidant activity. Therefore, the EO from *D. viscosa* could be employed as an antioxidant after more evaluations. Furthermore, the EO exhibited significant antimicrobial properties against nosocomial resistant microorganisms. The insecticidal efficacy of EO against the cowpea weevil (*C. maculatus*) is promising; therefore, this EO can be used as a natural bio-pesticide for the control of pests.

## Figures and Tables

**Table 1:** Chemical composition of the essential oil extracted from leaves of *D. viscosa*.

Peak	Compounds	Formula	CAS	RI Literature	RI Observed	RT *	EO (%)
1	α-Pinene	C_10_H_16_	7785-70-8	939	939	12.71	0.76
2	Isodrimenin	C_15_H_22_O_2_	13466-78-9	1942	1941	14.13	1.79
3	3-Carene	C_10_H_16_	13466-78-9	1011	1011	14.35	0.79
4	Santolina triene	C_10_H_16_	2153-66-4	908	907	14.61	0.28
5	Caryophyllene oxide	C_15_H_24_O	1139-30-6	1587	1587	16.12	5.73
6	Andrographolide	C_20_H_30_O_5_	5508-58-7	1674	1674	16.15	1.20
7	γ-Himachalene	C_15_H_24_	53111-25-4	1451	1450	17.16	0.45
8	τ-Muurolol	C_15_H_26_O	19435-97-3	1642	1642	17.56	1.09
9	τ-Cadinol	C_15_H_26_O	481-34-5	1640	1642	17.98	0.56
10	Camphene	C_10_H_16_	79-92-5	953	953	18.95	2.78
11	Epizonarene	C_15_H_24_	41702-63-0	1501	1500	19.35	0.90
12	Isoborneol	C_10_H_18_O	124-76-5	1160	1160	20.74	1.05
13	Farnesyl bromide	C_15_H_25_Br	28290-41-7	1902	1901	21.18	1.28
14	Fenchyl acetate	C_12_H_20_O_2_	4057-31-2	1220	1220	21.95	0.74
15	Borneol	C_10_H_18_O	507-70-0	1173	1170	23.15	9.33
16	Thujopsene	C_15_H_24_	470-40-6	1431	1431	23.35	2.25
17	Limonene	C_10_H_16_O	138-86-3	1029	1027	24.26	0.85
18	γ-Elemene	C_15_H_24_	29873-99-2	1432	1432	25.24	0.26
19	Isoledene	C_15_H_24_	95910-36-4	1374	1370	25.58	0.27
20	Caryophyllene	C_15_H_24_	87-44-5	1424	1420	26.23	0.68
21	Humulen-(v1)	C_15_H_24_	6753-98-6	1608	1608	26.58	0.42
22	Naphthalene	C_10_H_8_	91-20-3	1181	1180	27.68	3.25
23	Isoaromadendrene epoxide	C_15_H_24_O	22029-76-1	1460	1460	28.32	0.19
24	a-Bulnesene	C_10_H_8_	3691-12-1	1509	1508	28.74	1.00
25	Bicyclosesquiphellandrene	C_15_H_24_	54324-03-7	1522	1520	29.37	0.89
26	Spathulenol	C_15_H_24_O	523-47-7	1578	1578	30.04	1.19
27	bornyl acetate	C_12_H_20_O_2_	1617-68-1	1288	1280	31.32	41.00
28	Naphthalen-2-ol	C_10_H_8_O	93-0R-3	1447	1447	32.77	0.56
29	α-Cadinol	C_15_H_26_O	481-34-5	1654	1654	33.58	1.12
30	Ledol	C_15_H_26_O	577-27-5	1602	1600	34.18	1.09
31	11-Hexadecynal	C_15_H_24_O	86426-73-5	1503	1500	34.32	0.57
32	α-amorphene	C_15_H_24_	20085-19-2	1484	1480	35.02	6.60
33	Longifolenaldehyde	C_15_H_24_	19890-84-7	1614	1610	26.28	0.38
34	Aristolene epoxide	C_15_H_24_O	30824-67-0	1291	1290	37.14	0.44
35	Isoaromadendrene epoxide	C_15_H_24_O	7459-33-8	1641	1640	38.63	0.44
36	Aromadendrene oxide-(2)	C_15_H_24_O	28474-90-0	1628	1628	39.69	0.22
37	Caryophyllenol	C_15_H_24_O	4752-56-1	1572	1572	42.48	2.49
38	9-cis-Retinal	C_20_H_28_O	630-02-4	2800	2800	43.16	0.92
39	Verbenol	C_10_H_16_O	630-02-4	1141	1140	48.77	0.49
40	Pentacosane	C_25_H_52_	630-03-5	2500	2500	51.96	0.73
41	Lupan-3-ol, acetate	C_32_H_54_O_2_	7683-64-9	2815	2815	54.79	0.43
Total							97.46%

* RT: Retention time; RI: Retention index.

**Table 2:** IC_50_ (mg/mL) of the essential oil, BHT, and ascorbic acid by DPPH and FRAP assays. Results are expressed as mean ± SD.

	DPPH IC_50_ (mg/mL)	FRAP EC_50_ (mg/mL)
EOD	1.290 ± 0.055	35.585 ± 2.52
BHT	0.007 ± 0.001	1.256 ± 0.164
Ascorbic acid	0.001 ± 0.001	0.764 ± 0.125

**Table 3:** Total antioxidant capacity of the essential oil of *D. viscosa* dilution series in mg AAE/g E. Results are expressed as mean ± SD.

Dilution Series of Essential Oil							
	1/10	1/20	1/40	1/80	1/160	1/320	1/640
TAC (mg AAE/g E)	192.1 ± 0.8	190.1 ± 0.1	166.4 ± 0.6	152.8 ± 0.1	108.4 ± 0.4	77.2 ± 1.0	39.8 ± 0.7

**Table 4:** Disc diffusion test for essential oil from *D. viscosa* (EOD), performed on six pathogenic strains and using antibiotics for comparison. Inhibition zone diameter in mm. Results are expressed as mean ± SD.

Inhibition Zone Diameter (mm)					
Microorganisms	Antibiotics				
	EOD	Streptomycin	Tetracycline	Imazalil	Fluconazole
**Gram-negative**					
*E. coli*	9.5 ± 0.5	Resistant	18.5 ± 1.5	NA	NA
*P. aeruginosa*	Resistant	Resistant	13.2 ± 0.5	NA	NA
*K. pneumoniae*	Resistant	Resistant	15.0 ± 0.7	NA	NA
**Gram-positive**					
*S. aureus*	31.0 ± 1.5	9.5 ± 0.2	17.0 ± 1.2	NA	NA
**Fungus**					
*C. albicans*	20.4 ± 0.5	NA	NA	45.7 ± 1.2	21.0 ± 1.0
**Yeast**					
*S. Cerevisiae*	28.0 ± 1.0	NA	NA	47.0 ± 2.5	27.5 ± 0.5

NA: Not applicable.

**Table 5:** Minimum inhibitory concentrations (MIC) (mg/mL) of the EO extracted from *D. viscosa*. The comparison was performed with synthetic antibiotics.

Microorganisms	Minimal Inhibitory Concentration (mg/mL)				
		Antibiotics			
	EOD	Streptomycin	Tetracycline	Imazalil	Fluconazole
**Gram-negative**					
*E. coli*	0.406	0.250	0.250	NA	NA
*P. aeruginosa*	1.625	Resistant	0.250	NA	NA
*K. pneumoniae*	0.406	0.003	0.062	NA	NA
**Gram-positive**					
*S. aureus*	0.101	0.062	0.003	NA	NA
**Fungus**					
*C. albicans*	0.203	NA	NA	0.050	0.400
**Yeast**					
*S. cerevisiae*	3.250	NA	NA	0.010	0.200

NA: Not applicable.

**Table 6:** Lethal concentrations of *D. viscosa* EO against *C. maculatus.* Results are expressed as mean ± SD.

Treatment	LC_50_	LC_95_
Inhalation test	7.79 ± 0.29	14.36 ± 1.37
Ingestion test	14.46 ± 2.13	55.01 ± 8.46

**Table 7:** EO activity (at various doses) on eggs laid and emergence of *C. maculatus*. Results are expressed as mean ± SD.

Dose of EOD (µL)	Number of Eggs Laid	Reduction of Eggs Laid (%)	Percentage of Adult Emergence	Reduction of Emergence (%)
**Control (0)**	184.67 ± 23.43 ^a^	-	111.67 ± 6.51 ^a^	-
**1**	93.67 ± 9.61 ^ab^	49.28 ± 5.20 ^a^	75.33 ± 6.51 ^ab^	32.53 ± 5.82 ^ab^
**5**	65.0 ± 6.00 ^ab^	64.80 ± 3.24 ^ab^	51.67 ± 9.07 ^ab^	53.73 ± 8.12 ^a^
**10**	32.33 ± 7.02 ^abc^	82.49 ± 3.80 ^ab^	21.67 ± 6.11 ^abc^	80.59 ± 5.47 ^ab^
**20**	15.33 ± 3.51 ^abc^	91.69 ± 1.90 ^ab^	9.67 ± 2.31 ^ab^	91.34 ± 2.06 ^ab^

Column values with the same letter differed significantly (*p* < 0.05 (Tukey’s HSD test). The results are presented as mean ± SD. All treatments had a significant effect compared with the control (*F* = 5.09, *p* = 0.016).

## Data Availability

All data reported here is available from the authors upon request.

## Sample Availability

Samples of the compounds are available from the authors.
